# Induced Premature Labour in Certain Diseases of the Mother Not Obstructing Delivery

**Published:** 1896-03

**Authors:** J. G. Swayne

**Affiliations:** Emeritus Professor of Midwifery, University College, Bristol; Consulting Physician-Accoucheur, Bristol General Hospital


### INDUCED PREMATURE LABOUR IN CERTAIN DISEASES OF THE MOTHER NOT OBSTRUCTING DELIVERY.

BY

J. G. Swayne, M.D. Lond.,

*Emeritus Professor of Midwifery, University College, Bristol; Consulting Physician-Accoucheur, Bristol General Hospital.*

---

During a practice of rather more than half a century, I have met with a number of cases requiring the induction of premature labour. In the present paper I propose to describe more particularly the diagnosis, symptoms, and treatment of cases, which illustrate the "necessity of inducing labour in certain diseases of the mother *not obstructing delivery*."[1] Since I have been in practice I have carefully recorded a total number of thirty-seven cases in which I found it necessary to induce premature labour. In twenty-four of these I had to do so on account of *strictly mechanical* obstruction to delivery, arising in nearly all from pelvic deformity. After making this deduction, thirteen cases are left, in which the continuance of pregnancy was rendered undesirable and dangerous by the presence of certain diseases and complications due solely to pregnancy, and not to any *obstruction* likely to cause difficult and dangerous labour.

Dr. Robert Barnes's masterly work, *Lectures on Obstetric Operations*, first published in 1870, has been to me a most useful guide and counsellor in obstetric difficulties of this kind. On page 378 of the fourth edition, 1886, Dr. Barnes gives a list of "certain cases of urgent disease of the mother, depending upon and complicating pregnancy—*e.g.*, obstinate vomiting, with progressive emaciation, and a pulse persistent

[1] This was one of the subjects for discussion in the Section of Obstetrics at the Bristol meeting of the British Medical Association in 1894. It was introduced by Dr. Robert Barnes. As President of that Section, I had little opportunity to do more than enumerate my cases.

for some days above 120; some cases of advancing jaundice, with diarrhœa; some cases of albuminuria, convulsions being present or apprehended; some cases of insanity or of chorea; hæmorrhages producing marked anæmia, especially if depending upon commencing abortion or placenta prævia; some cases of disease of the heart and lungs, attended with extreme dyspnœa; such are aneurism, great hypertrophy, valvular disease, œdema of the lungs, pleurisy." I propose now to go through the above list of maladies which necessitate the induction of premature labour, giving, as I proceed, illustrations of each from my own cases, together with a few comments. The first on the list is *obstinate vomiting*. This may appear at first to be nothing more than a morbid exaggeration of the ordinary morning sickness of pregnancy. But it is the most common and often the most obstinate of these affections, for it will frequently defy all other remedies but that of cutting short the pregnancy. My own statistics show that it is the most common, for no less than five out of my thirteen exceptional cases were of this kind. Moreover, it often causes more anxiety to the medical attendants than any other, for its dangerous nature is shown by the fact that in these five cases two mothers and three infants lost their lives. That it involves an excessive amount of responsibility arises from the circumstance that most of these cases, *e.g.* three out of the five, came on before the infant was viable, and therefore if not already dead, its life had to be sacrificed to save that of the mother. And this very fact adds very much to the risk of such labours. Every conscientious practitioner naturally feels the greatest repugnance to destroying the life of the fœtus by inducing abortion, and therefore postpones this disagreeable necessity as long as possible, too long sometimes to be compatible with the safety of the mother—as, for instance, when she is very nearly at starvation point, when irritative fever has ensued, and the pulse has risen persistently to 140 or more. When things have come to this pass, as Dr. Barnes remarks, "no organ in the body is capable of discharging its functions, for the pabulum of life is cut off at its very source. At this point labour, whether it occur spontaneously, as it often does, or be brought on artificially, comes too late. The tissues are altered, the powers are impaired beyond recovery, and death soon follows delivery." The first of my five cases of obstinate vomiting was unfortunately one of this kind.

Case 1.—On December 3rd, 1862, Mr. Crossman requested me to see Mrs. A., a patient of his in the country at Mangotsfield, who was within a few days of her first confinement. For more than a week she had been suffering from obstinate vomiting, which no medicine would allay, accompanied with excruciating pain in the epigastrium on taking the smallest particle of food, and also the ejection of quantities of grumous blood, which made me suspect that a gastric ulcer was present. She could keep down but small particles of ice, and was in a state of extreme prostration. After a short consultation we both decided that the sooner labour was induced the better. On examination I found that I could just reach the os uteri and insert the tip of my forefinger in it, but it was high up and far back. There had been no labour pains of any kind. Wishing to bring on labour as quickly as possible, about 7 p.m. the same evening I punctured the membranes with a uterine sound, and let out a good deal of liquor amnii. We also injected into the rectum ℥ss. of the ethereal tincture of ergot with a little gruel. Labour pains soon came on and returned regularly. During the labour we kept up her strength by giving small quantities of frozen cream, and also brandy. About 5.30 a.m. December 4th, she gave birth, after a ten hours' labour, to a living and tolerably vigorous male child. Soon after this there was some *post-partum* hemorrhage. It was a very moderate amount and was easily checked. Still, it left her in a state of extreme prostration. However, she had rallied considerably about three hours afterwards, when I left her in charge of Mr. Crossman and returned home. She went on tolerably well until December 7th, taking a fair amount of food and stimulants without rejecting any. On December 8th she was attacked with diarrhœa, which was not very severe, but depressed her very much, although it had been much checked during the two following days; but on December 10th, about 8 p.m., she fainted suddenly, did not rally, and died about 10 o'clock that evening.

The unfortunate issue of this case was very disappointing, and more to be lamented because everything appeared so favourable on the first two or three days after delivery. Her death, I think, was mainly due to increased prostration caused by the diarrhœa which set in on the fourth day, and added to the depression already produced by the very moderate amount of *post-partum* hemorrhage.

The next case has much resemblance to the last in its earlier features, but is on the whole more interesting and much more satisfactory in every way.

Case 2.—On March 2nd, 1867, Mr. Burleigh requested a consultation with me on the case of Mrs. B., living in Stokes Croft, Bristol. She was a primipara about eight months pregnant, who had suffered lately from incessant vomiting, which had defied all the usual remedies. This had become much worse during the night preceding, when she was in a most prostrate condition, at times almost pulseless, and constantly throwing up much coffee-ground vomit. When I saw her she was very little better, for the vomiting was urgent and the prostration excessive. There was a good deal of epigastric tenderness, and in the right epigastric region I felt a pedunculated tumour about the size of an egg, movable and very tender. This was attached to the fundus uteri and had probably by its pressure produced the gastric disturbance. We both considered that no time was to be lost, and decided on immediately inducing labour. On examining, I found the os uteri dilated to the size of a shilling, although there had been no labour pains, and I was able to feel an elbow presenting. I then began to dilate the os with one of Dr. Barnes's caoutchouc bags of the smallest size, but in so doing the membranes gave way. As the os uteri was now tolerably dilatable, I determined to attempt version by Dr. Braxton Hicks's bimanual method. This I effected without much difficulty. Having pushed up the elbow, I was soon able to grasp a foot which came down, and, before long, to extract the child, which was a male, alive and apparently vigorous for an eight months' child. It died, however, four days after delivery. The mother made a good recovery, the pedunculated fibroid descending with the uterus and giving no further trouble. The delivery occupied only six hours.

In these two cases it would be difficult to say, before labour commenced, which woman was in the worse condition. The depression was so extreme that life, in either case, seemed to hang on a thread. In the second case, fortunately no after-complications occurred as in the first, and the complete success which followed delivery was the best argument in favour of adopting this remedy when all others had failed. The presence of a tender pedunculated tumour growing from the fundus uteri and pressing on the stomach might possibly have tempted an enterprising young surgeon, with little obstetric experience, but much skill in abdominal surgery, to remove by a simple abdominal operation what was apparently the cause of all the mischief. This, however, would have inflicted an additional shock on a system too feeble to bear it, and might have failed in the result, and so might in the end have rendered it necessary to adopt the induction of labour as a last resource and under the most disadvantageous circumstances. Fortunately for the mothers, especially in the last case, the infants had both passed the seventh month of pregnancy, and were therefore viable. We had accordingly no conscientious scruples on *their* account to prevent us from expediting the delivery.

This, however, was not the case in the next example of obstinate vomiting which I had to treat.

Case 3.—On August 19th, 1871, Mr. Davies of Gloucester Road requested me to see Mrs. C., of West Street, Bristol, for most obstinate vomiting during early pregnancy. She had suffered very much from vomiting during her two previous pregnancies, but had gone her full time on each occasion. This time, however, the vomiting had been very violent, and had resisted most of the usual remedies such as prussic acid, oxalate of cerium, creasote, and morphia both internally and hypodermically. The vomiting returned every half hour, and she had scarcely kept down anything for several days, and was rapidly becoming more weak and exhausted. She was now about 3½ months pregnant. We first of all agreed to try the effect of keeping open a blister on the epigastrium and applying half a grain of morphia endermically. She was slightly better on the next day from this treatment, but on the day following there was no improvement, and as her case was becoming very critical, we determined to induce abortion. Accordingly, I introduced into the os uteri, through a speculum, a rather large tangle tent. On the next day this had induced no pains, but the os uteri was only enough dilated to admit the tip of the forefinger. I then introduced a tent of still larger size. About 7 p.m. on the same evening, Mr. Davies was sent for because she had some vaginal hemorrhage, together with labour pains. On arriving, he removed several clots and with them a dead fœtus of between three and four months' growth, and also a portion of placenta. On the next day I removed the remainder of the latter by means of an ovum forceps. The vomiting had much abated by that time, and before long had entirely ceased, and the patient did well.

In this last case, after giving all sorts of medicine and putting off the evil hour as long as possible, we were at last driven to bring on abortion to save the mother. I will give reasons for adopting this course, when I come to speak of another, but a still worse case, during still more early pregnancy (Case 5).

The next case was a very painful one, for the unfavourable result was mainly due to the patient's own fault:

Case 4.—Dr. Logan of Bedminster, Bristol, called me in to see Mrs. D., a multipara, residing in Bedminster. She had previously had seven children. Her former labours had been favourable and also her pregnancies. Unfortunately, during the last two years, in consequence of domestic trouble, she fell into a low, weak and hysterical condition; and to relieve such feelings had taken to drink immoderately, and in consequence had suffered from intense vomiting during the present pregnancy. I was first asked to see her on January 26th, 1877, when she was rather more than four months pregnant. I ordered her a mixture containing bismuth and creasote, and this had for a time a very good effect. But before two months were over the vomiting returned with increased violence, and she became so much worse that I was requested to see her again on March 16th, 1877, when she was rather more than six months pregnant. The vomiting was then almost incessant, and she brought up large quantities of bile of a grass-green colour and could scarcely retain any food. Before seeing her, I had on the 11th of March ordered her to resume the bismuth and creasote mixture, and to apply a blister to the epigastrium and afterwards half a grain of morphia to the blistered surface. This caused relief for a time, but on March 16th I found that the vomiting had returned with increased violence, accompanied with increased exhaustion. Her condition was so precarious that, although the child was not quite viable, we decided to bring on labour without delay. About 11 a.m., March 16th, I introduced a large sponge tent, for I found the os uteri dilated enough to admit two fingers and the head, covered by the membranes, presenting. I was called up during the night, about 1 a.m., when Dr. Logan told me that he had removed the tent because labour pains were coming on rapidly—so much so, I found, that about 2.45 a.m. she gave birth to a still-born infant of between six and seven months' development. The labour was followed by great debility, without any apparent cause, which increased so rapidly that she died about 5 a.m. on the following day.

On reviewing this case there can be, I think, no doubt that the intemperate habits to which the patient had lately given way were the principal cause of the fatal termination. When I began to induce labour I found the os uteri already open enough to admit two fingers, just as if labour had commenced already; and the child, when born, looked as if it had been dead for some hours—just as if Nature had made one last expiring effort to promote its expulsion.

In Dr. Barnes's[1] address at the Bristol Meeting there is an excellent passage so applicable to this case that I cannot refrain from quoting it. "Vomiting," he says, "is most severe when it sets in with severe mental shock or distress; when there is gloomy depression passing into despondency. Such cases, may run rapidly to a fatal issue, resisting all treatment even the induction of labour. Toxæmia soon complicates the case, even when the embryo lives. First, there is starvation giving strong impetus to the process of absorption; secondly, no adequate nutrition being supplied from without, the system feeds upon itself. There is rapid disintegration of tissue, most marked in the fat; so the blood is degraded and empoisoned. . . . At this point labour, whether it occur spontaneously, as it often does, or be induced artificially, comes too late. Death soon follows delivery."

My last case of this kind was an example of vomiting of

[1] *Brit. M. J.*, 1894, ii. 1419.

the most obstinate and irrepressible kind, occurring in early pregnancy.

Case 5.—On February 6th, 1891, Dr. Long Fox and Dr. Aust Lawrence asked me to consult with them on the case of Mrs. E. She reckoned herself to be about three months advanced in her first pregnancy, but for more than a month had suffered from the most urgent vomiting, which had resisted all the usual remedies, so that she could not keep down even milk and lime-water nor any of the various peptonised foods. A few days before I saw her, Dr. Fox had consulted with Dr. Lawrence, who tried, with apparently good effect at first, Dr. Copeman's plan of dilating the os uteri and using for that purpose Hegar's dilators. But as there was no well-marked improvement, I was consulted as to the advisability of ultimately inducing abortion. When I saw her I found her emaciated to an extreme degree; pulse about 115; temperature a little above normal; vomiting not quite so urgent; uterus anteverted more than usual during early pregnancy, with a rather sharp bend at the os uteri internum. As there was some mitigation of the sickness, we decided to wait a day or two longer before resorting to such an extreme and responsible step as the induction of abortion; we, therefore, ordered an opiate enema. On the next day we found that she had slept a little better and could keep down a little milk. On the day following, however, Dr. Fox and I found her much worse; she had passed a sleepless night; vomiting very urgent; pulse 120 and occasionally intermitting; temperature 97° ; tongue, dry and brown. Unfortunately, Dr. Lawrence was prevented from meeting us by another engagement at a distance, and her condition was so anxious that we both agreed that labour should be brought on without delay. I therefore passed, completely into the uterus, a medium-sized tangle tent, which I had previously moistened and bent to the right angle to enable it to pass the sharp curve in the cervix uteri. This I left *in situ* for twenty-four hours. On calling the next morning we found that the tent had dilated the uterine neck and straightened it, and that there was some show, but no labour pains; the other symptoms, if anything, not so urgent. I therefore inserted a larger tent, which I also left *in situ* twenty-four hours. On the next day, February 10th, I found that there had been more show, but no labour pains. The os, however, was fairly dilated. I then introduced a tolerably large sponge tent, intending to leave it for twelve hours; but I had not been absent more than eight hours when I received an urgent message from her husband, stating that she had had a succession of sharp pains with a good deal of bloody discharge. On arriving at the house he told me that the sponge tent had been expelled and also the fœtus, which I saw. It was about an inch long and had a flattened, collapsed and macerated appearance as if it had been dead for some days. As no more of the ovum had been expelled, I introduced two fingers and removed the secundines, which had nearly passed through the os uteri. The membranes were rather thickened, and the placenta was not quite fully formed. From the development, I should consider that the ovum had not quite reached three months' growth. I then gave her an opiate and washed out the vagina with warm water and Condy's fluid. On the next day I found that she had passed a good night, that there had been no more vomiting, and that her appetite was returning. Pulse 104; temperature normal. From that time she soon got better, and made a good recovery; but it was a long time before she again regained her strength.

This case was one of unusual urgency at so early a period of pregnancy. It is therefore important, for it impresses on one's mind the fact that at such times "delays are dangerous." The state of the fœtus when expelled appeared to show that the extremely critical condition of the mother, who may truly be said to have arrived at "death's door" when this happened, had already, some days previously, destroyed the vitality of the embryo. And thus from a laudable desire to save the frail life of the embryo, which is probably already dead, one *may* be tempted to defer active measures too long, and at last lose the life of the mother also. As Dr. Barnes most judiciously remarks, "We must be careful not to be deluded into pursuing the policy of expectancy too far. The transition from physiology to pathology is often insidious or sudden." And again, "The strictly medical treatment of the difficulties that complicate pregnancy is often delusive. We must not defer too long an appeal to the decisive intervention of surgery."

On taking a retrospective glance at my notes of these five cases, I cannot help thinking that, in Nos. 1 and 4, an earlier interference of a decisive kind, in accordance with this excellent advice of Dr. Barnes, might possibly have secured a better result for the mothers. This refers especially to No. 1, in which case the infant was saved, because it was not only viable but very near the full term. But in most of the cases like those I have related, there are two evils to be avoided, a greater and a less; the former being the death of the mother, and the latter of the infant. They stand like two rocks ahead, either of which is to be avoided if possible, but especially the greater. And to steer clear of both will require the combined skill and experience of at least two thoroughly competent pilots.

(*To be continued.*)

